# Reduction in Predator Defense in the Presence of Neighbors in a Colonial Fish

**DOI:** 10.1371/journal.pone.0035833

**Published:** 2012-05-16

**Authors:** Franziska C. Schädelin, Stefan Fischer, Richard H. Wagner

**Affiliations:** 1 Konrad Lorenz Institute of Ethology, Department of Integrative Biology and Evolution, University of Veterinary Medicine, Vienna, Savoyenstrasse, Vienna, Austria; 2 Institute of Ecology and Evolution, University Bern, Wohlenstrasse, Hinterkappelen, Switzerland; University of Utah, United States of America

## Abstract

Predation pressure has long been considered a leading explanation of colonies, where close neighbors may reduce predation via dilution, alarming or group predator attacks. Attacking predators may be costly in terms of energy and survival, leading to the question of how neighbors contribute to predator deterrence in relationship to each other. Two hypotheses explaining the relative efforts made by neighbors are byproduct-mutualism, which occurs when breeders inadvertently attack predators by defending their nests, and reciprocity, which occurs when breeders deliberately exchange predator defense efforts with neighbors. Most studies investigating group nest defense have been performed with birds. However, colonial fish may constitute a more practical model system for an experimental approach because of the greater ability of researchers to manipulate their environment. We investigated in the colonial fish, *Neolamprologus caudopunctatus,* whether prospecting pairs preferred to breed near conspecifics or solitarily, and how breeders invested in anti-predator defense in relation to neighbors. In a simple choice test, prospecting pairs selected breeding sites close to neighbors versus a solitary site. Predators were then sequentially presented to the newly established test pairs, the previously established stimulus pairs or in between the two pairs. Test pairs attacked the predator eight times more frequently when they were presented on their non-neighbor side compared to between the two breeding sites, where stimulus pairs maintained high attack rates. Thus, by joining an established pair, test pairs were able to reduce their anti-predator efforts near neighbors, at no apparent cost to the stimulus pairs. These findings are unlikely to be explained by reciprocity or byproduct-mutualism. Our results instead suggest a commensal relationship in which new pairs exploit the high anti-predator efforts of established pairs, which invest similarly with or without neighbors. Further studies are needed to determine the scope of commensalism as an anti-predator strategy in colonial animals.

## Introduction

Predation of offspring has long been stressed as one of the major factors affecting the fitness of breeders [1], [2]. This factor has spawned many studies about behavioral strategies of nest defense, which often comprise direct attacks or approaches by breeders to drive away predators. Such nest defense is costly in terms of time and energy [2], and may even result in the death of defenders [3], [4]. Proposed ways to reduce the costs is to breed in close proximity to multiple neighbors, which allows breeders to benefit from earlier detection, dilution of predation and by group defense [5], [6]. At a theoretical level, cost/benefit models have long been applied to identify optimal investment in nest defense [7]–[14]. Empirically, a number of field studies have examined group defense by placing predators or models near breeding sites and observing the relative predator attack rates of neighbors. In tree swallows *Tachycineta tricolor* for example, multiple neighbors mobbed predators placed near their nests, thereby increasing the intensity of predator defense per nest [15]. This effect was also shown in colonial Montagu’s harriers *Circus pygargus* in which the probability of the predator model being attacked increased with group size [16]. Furthermore, the individual rate of high-risk diving-attacks decreased with group size, suggesting that breeding near close neighbors also reduces the individual net costs of defense [16].

Two hypotheses are currently debated to explain the relative contribution of predator defense by neighbors [17]–[19]. The first hypothesis is byproduct mutualism [20], which occurs when, by defending their own nest, breeders inadvertently chase predators away from close neighbors. The second hypothesis is reciprocity, which occurs when breeders deliberately exchange predator defense efforts with neighbors [21]. This debate was stimulated by an experiment in which focal pairs of pied flycatchers *Ficedula hypoleuca* where given the option of attacking either of two owl predator models placed near the nests of two neighboring pairs. One pair had previously joined the focal pair in attacking a predator at their nest whereas the other pair had been prevented from doing so [22]. Focal pairs assisted the pair that had previously joined them in defense in 30 of 32 trials whereas they never assisted the pair that had not previously assisted them [22]. The authors, as well as a subsequent commentary [19], suggested this finding to be strong evidence of reciprocity, although other authors proposed that it could be explained more parsimoniously by byproduct mutualism [18].

Both hypotheses are components of the reduced predation hypothesis, which has long been proposed as a leading explanation of colonial breeding. Despite decades of research, evidence for the reduced predation hypothesis remains mixed, with some studies supporting it [16], [23]–[28], and other studies suggesting that colonies attract predators [5], [29]. Nearly all studies have been performed with birds. Certain fish species however, may provide an alternative model system to further examine nest defense strategies because fish allow researchers to more easily manipulate the environment. We studied a biparental, colonial cichlid fish to examine (1) the degree to which prospecting pairs prefer to breed near an established pair versus solitarily, and (2) the relative investment in predator deterrence between two close neighbors.

## Methods

### The Study Species

*Neolamprologus caudopunctatus* is a member of the species-rich tribe Lamprologini [30]–[32], which accounts for ca. 40% of the ∼300 cichlid species in Lake Tanganyika [33], [34].

The breeding system of *N. caudopunctatus* shares a number of salient features with colonial birds, especially seabirds, such as (1) sexual monomorphism in shape and color, (2) biparental care [35], and (3) breeding in dense aggregations. We have observed *N. caudopunctatus* in Lake Tanganyika breeding in colonies of more than 100 pairs as well as in smaller groups of 5–10 pairs, with a mean nearest neighbor distance of less than one meter. The monogamous mates transfer sand to build a breeding cavity under stones, in rock crevices or gastropod shells, in which the eggs are laid. Both parents defend the breeding cavity containing eggs and larvae and subsequently defend free swimming fry which remain in close proximity to the cavity.

Juvenile *N. caudopunctus* are apparently under severe predation pressure. Ochi [35] identified 29 cichlid species that regularly prey on *N. caudopunctatus* offspring. Most predators pose a risk only to larvae and free swimming fry, however some, such as *L. elongatus,* also predate adults. Parents attacked potential predators more than 12 times per ten minutes and broods without brood caring parents were usually predated, sometimes within one minute after the removal of the parents [35].

For our experiment we used wild *N. caudopunctatus* caught in Lake Tanganyika near Mpulungu, Zambia, Africa. *N. caudopunctatus* is an endemic cichlid to Lake Tanganyika feeding exclusively on plankton [35]. Males attain a mean total length (TL) of 7.5 cm and females of 6.5 cm. We measured standard length (SL), from the tip of the longest jaw to the end of the base of the caudal fin, total length (TL), height 7.5 cm (H) and weight (W) of each fish before the experiment. Throughout the experiment the fish were fed daily with frozen food (Artemia sp., Cyclops sp., red mosquito larvae and Daphnia sp.) and with flakes for tropical fish, and kept at a constant water temperature of 27±1°C under a 13/11 h day/night cycle.

### Ethical Note

The authors manipulated and marked *N. caudopunctatus* under the following animal permits from the Austrian Federal Government Department for Science and Research: BMWF-66.015/0016-II/10b/2009 according to the Austrian law TVG, BGBI.Nr. 501/1989, last changed by BGBI. I Nr. 162/2005.

### Experiment

**Figure 1:**
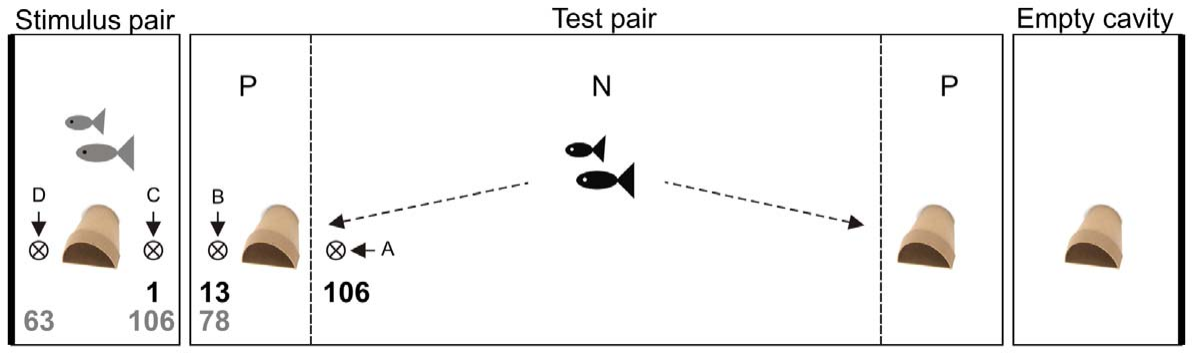
The experimental set-up. One large central tank for the test pair and two adjacent small tanks comprised the experimental set-up. The two small tanks each contained a potential breeding cavity (flower pot), one occupied by a stimulus pair and the other unoccupied. The large central tank contained two potential breeding cavities and a test pair. A, B, C and D indicate the two neighbor (B, C) and two non-neighbor (A, D) predator presentation sites. To assess the location of the test pair we divided the central aquarium into two preference zones (P) and one neutral zone (N). The numbers below the presentation sites are the median attack rates of the pairs at the respective location. Gray numbers are the anti-predator attack rates by the stimulus pair and black numbers are the attack rates by the test pair.

The experimental setup consisted of a central 400l aquarium and two adjacent small side tanks (50l), all with a 2−3 cm sand layer on the floor and half flower pots as potential breeding cavities (Fig. 1). We started each trial by forming a stimulus pair, which entailed placing together an unfamiliar male and female into one of the two side tanks and allowing them to bond and construct a breeding cavity. After three days, we tested whether the male and the female had pair-bonded by presenting them with small intruders in a transparent plastic tube directly in front of their breeding cavity. We used young *N. caudopunctatus* (sized between 1.5 cm and 2 cm TL) as intruders, which are conspecific egg predators. Each intruder test lasted for five minutes, including two minutes acclimatization and three minutes observation. We recorded the frequency of the attack rates by the male or female and by the pair attacking simultaneously, and considered pair formation to have been established following at least five attacks by both partners. If we observed no attacks after six days we terminated the trial and began a new trial with another potential stimulus pair, which had occurred in one of 15 cases. We alternated the two side tanks between trials to account for possible side preferences.

Simultaneously to the introduction of the stimulus pair, we introduced a male and female comprising the future test pair into a separate tank in another room and allowed them to form a pair bond. After both the test and the stimulus pairs formed, we released the test pair in the middle of the central aquarium. Although both pairs had equal time to bond, stimulus pairs had more time to invest in territory establishment before the experiment started. In the central aquarium, the test pair was presented with two potential breeding cavities, one next to the stimulus pair and one next to the opposite, uninhabited side tank (Fig. 1).

After 14 days (or after egg-laying, if it occurred earlier) we determined the breeding site choice of the test pair using three criteria. First, we measured the building activity using six different stages, ranging from 0 (no building activity) to 5 (flower pot filled with sand to capacity). Second, during intruder presentations we observed the attack rates in front of both potential breeding sites. Third, we divided the central tank into three zones: two preference zones, next to the two side tanks (each 21.5 cm×62 cm) and one neutral zone, between the two preference zones (82.5 cm×62 cm; Fig. 1) and measured the time that each pair member spent in one of the two preference zones. The duration of this experiment was three minutes including one minute of acclimatization and two minutes of observation. We further recorded the location of egg-laying whenever it occurred in either of the two potential breeding sites.

To elicit nest defense behaviors, we presented three small predators (*Lepidiolamprologus elongatus,* size 4 to 5 cm TL) inside a transparent plastic tube. To compare the intensity of anti-predation attacks by one pair versus both pairs simultaneously (i.e. group defense), we placed the presentation tube at four different positions in each trial (Fig. 1): (1) inside the central tank next to the test pair, but far from the stimulus pair (position A), (2) between the stimulus pair and the test pair inside the central tank (position B), (3) between the stimulus and the test pair within the side tank (position C) and (4) inside the side tank next to the stimulus pair, but far from the test pair (position D). Each presentation lasted ten minutes and was recorded using digital video cameras. Afterwards videos were analyzed using “The Observer^©^ XT 7.0”. We tabulated all behaviors observed: fin spreading, approach (approaching the presentation tube without contact), attack (approaching the presentation tube with contact or where this was impossible due to the tank wall, contact with the tank wall), bars (changing skin color) and head down (swimming in a head down position). We also noted whether the two aggressive behaviors “approach” and “attacks” were targeted against the neighboring pair or against the presented predators.

The statistical analyses were performed using SPSS. All data were tested for normality with the Shapiro-Wilk test. For non-normally distributed data, non-parametric statistics were used. We used the Wilkoxon-matched-pair signed-rank test for dependent data.

## Results

Of 15 test pairs, 13 settled near the stimulus pair and two settled near the unoccupied breeding site (binominal test, p = 0.007). Settlement location was confirmed by our three criteria: (1) showing higher building activity (N = 15, near stimulus pair: median = 4.00; near unoccupied breeding site: median = 1.00; Wilcoxon Z = –2.69, p = 0.007), (2) attacking intruders more frequently (N = 15, near stimulus pair: median = 57.00; near unoccupied breeding site: median = 6.00; Wilcoxon Z = –2.84, p = 0.005) and (3) spending more time at the settled location (N = 15, near stimulus pair: median = 133.58; near unoccupied breeding site: median = 0.00; Wilcoxon Z = –3.24, p = 0.001). Of the 11 test pairs that laid eggs, all did so inside the breeding site where they had settled according to the three criteria.

For the analyses of the predator presentation, we focused on attacks because they were the most unambiguous behavior identified as nest defense and intruder deterrence. In contrast to the other behaviors, attack was never observed in contexts other than aggression and the target of attacks was unambiguous.

**Figure 2:**
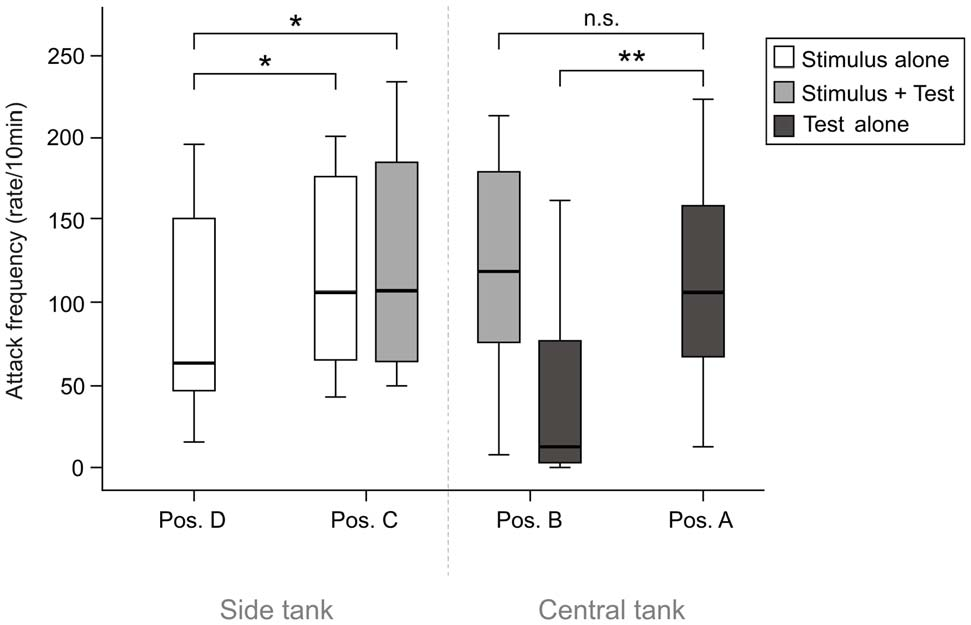
Anti-predator attack rates by test and stimulus pairs at their neighbor and non-neighbor positions. Test pairs decreased their attack rates next to their neighbor. Stimulus pairs increased their attack rates next to the neighbor. Position A: predator presentation on the non-neighbor side of the test pair; Position B: intruder presentation on the neighbor side of the test pair; Position C: intruder presentation on the neighbor side of the stimulus pair; Position D: intruder presentation on the non-neighbor side of the stimulus pair. *P<0.05, **P<0.001. In dark grey the attack rate of the test pair; in light grey the combined attack rate of both pairs; in white the attack rate of the stimulus pair.

When we presented predators to test pairs that had settled near the stimulus pair, their attack rates were approximately eight times higher at the far side of the breeding site than at the side neighboring the stimulus pair (Fig. 2; position A vs. B: N = 11, Wilcoxon Z = –2.93, p = 0.003). In contrast, the combined attack rates of test and stimulus pairs at positions A and B did not differ significantly (N = 11, Wilcoxon Z = –0.49, p = 0.62, Fig. 2). We also performed predator presentations to both sides of stimulus pairs’ breeding sites and found a significantly higher attack rate on the side neighboring the test pairs (Position C vs. D; N = 11, Wilcoxon Z = –2.14, p = 0.033). This finding remained the same after combining the attack rates of the test and stimulus pairs (Position C vs. D; N = 11, Wilcoxon Z = –2.13, p = 0.033).

We considered whether conspecific aggression between neighbors may influence the willingness to attack the presented predators. There was no significant correlation between the attack rate on neighbors and the attack rate on predators presented between the nest sites (Position B: N = 11; Spearman rho = –0.080; p = 0.816; Position C: N = 11; Spearman rho = –0.153; p = 0.654).

## Discussion

Our study has produced two main results. First, pairs of the biparental fish, *N. caudopunctatus,* strongly preferred to breed near neighbors in a simple choice test, which is consistent with their prevalence of colonial breeding in nature. Second, test pairs reduced their anti-predator attack rates eight-fold when settled near an established pair relative to the non-neighbor side of their breeding site (Fig. 2, Position B vs. position A). We first examine potential explanations for the observed conspecific attraction and then discuss several explanations for the greatly reduced attack rate by the test pairs.

We consider conspecific attraction in our study species in relation to four major hypotheses of colony formation. First, the information center hypothesis proposes that breeders settle near neighbors in order to follow them to unpredictable food sources [36]. This hypothesis can be excluded because breeders do not provide information on the location of food, which comprises plankton that drift through the water column in their breeding areas.

Second, the hidden lek hypothesis predicts that males settle near other males in order to obtain mates that seek extra pair fertilizations from neighboring males [37], [38]. This is unlikely in *N. caudopunctatus* because their ability to obtain all their food at the breeding site results in continuous nest and mate-guarding and in the protection of the narrow entrances of their breeding caves. Furthermore, our unpublished DNA analyses of almost 300 fry revealed no cases of extra-pair fertilizations.

Third, the habitat copying hypothesis predicts that prospectors choose breeding sites based on the performance of conspecifics [39]. Our finding is consistent with this prediction in that the presence of an established pair with a well built breeding cavity could indicate better breeding habitat there than at the unoccupied site. Thus “public” [40] or “performance” information [41] produced by the stimulus pair might explain conspecific attraction in the first stage of the experiment. However in the second stage, habitat copying cannot explain the reduced attack rate by the test pair.

Finally, conspecific attraction could be explained by the reduced predation hypothesis because by settling near close neighbors, breeders may obtain greater safety through increased mobbing [2]. Results in some studies have been interpreted as involving reciprocity or byproduct mutualism in predator defense among neighbors [22], [42]–[44]. If either reciprocity or byproduct mutualism occurred in our predator presentations, both pairs would have reduced their effort in both locations between their breeding sites (Fig. 1, locations B & C). Instead, there was a large reduction in attack rates by the test pair and no reduction by the stimulus pair.

An explanation for the asymmetric attack rates may be that later settling breeders join established breeders to exploit the latter’s predator deterrence effort. Doing so may benefit the test pair while incurring few or no costs to the stimulus pair, given that it would need to defend its breeding site also in the absence of a neighbor. This should be equally true for the more recently established test pairs on their non-neighbor sides, which was the case. The median rate of 106 attacks/10 min by the stimulus pair at position C was identical to that of the test pair on its non-neighbor side (position A). These patterns suggest a strategy of commensalism whereby the high attack rates of the stimulus pairs allow the test pairs to reduce their effort at position B. Alternatively, it is possible that the reduced attack rates by the test pair were caused by conspecific aggression by the stimulus pair, which might deter the test pair from attacking the shared predator. However, there was no relationship between the conspecific attack rate and the relative attack rate of the pairs towards the predators.

The attack rate at position D was substantially lower than at C (63 versus 106), which is inconsistent with the idea that the observed patterns are produced by a strategy of commensalism where a similar attack rate on the stimulus pairs’ non-neighbor side is expected. The reasons for this lower than expected rate is unclear but it may be an artifact of the large difference in tank size between the two pairs. Our set-up of the settling preference test required providing the test pair with a much longer tank then the stimulus pair. It is possible that the test pair might perceive the additional space in its larger tank as part of its territory. This possibility is supported by the observation in an other study that pairs often incorporated additional breeding sites into their territory when kept in a large 16,000 liter tank with many unoccupied breeding sites [45]. The difference in tank size and numbers of breeding sites might thus explain the lower attack rates by the stimulus than the test pairs on their non-neighbor sides.

To our knowledge this is the first experimental study of predator defense in a colonial fish, in which the environment can be more easily manipulated than in birds. Our study suggests that conspecific attraction in settling near neighbors for enhanced predator deterrence is not necessarily explained by reciprocity or byproduct mutualism. Alternatively, prospective breeders may exploit established breeders that may maintain a high predator deterrence effort regardless of the presence of neighbors. Further studies of predator defense may elucidate whether animals widely pursue a strategy of commensalism in safe-guarding their offspring.
